# Expanding the clinical spectrum: A case report of the first Jordanian presentation of KID syndrome with neurological and skeletal anomalies beyond the classical triad

**DOI:** 10.1097/MD.0000000000045577

**Published:** 2025-11-21

**Authors:** Rama Al-Bustanji, Bayan K. AlRababah, Miral S. Abu Rumman, Yara Abukhaled, Nizar Al-Rabadi, Hamzeh K. Bany Younis, Gharam Ghalyon, Safaa Al-Tawalbeh, Osama Aloudat, Mu‘nis Muneeb Mohammad Alrashdan, Mohannad Thafer Yamin, Muhanad Maaita, Aya Khaled D. Salah, Anas Satari

**Affiliations:** a Faculty of Medicine, Mutah University, Al-Karak, Jordan; b Jordan University of Science and Technology, Irbid, Jordan; c Faculty of Medicine, University of Jordan, Amman, Jordan; d Department of Internal Medicine, The University of Tennessee Health and Science Center, Memphis, TN; e Department of General Pediatrics, King Talal Military Hospital (JRMS), Al-Mafraq, Jordan; f Department of Medicine, Prince Hamza Hospital, Amman, Jordan; g Department of Neuropediatrics, King Hussein Medical Center, Amman, Jordan; h Department of Pediatrics, Saint Louis University School of Medicine, SSM Health Cardinal Glennon Children’s Hospital, St. Louis, MO; i Faculty of Medicine, Mutah University, Al-Karak, Jordan; j Depratment of Internal Medicine, King Hussein Cancer Ceter, Amman, Jordan; k Department of General Pediatrics, Maternity and Children’s Hospital at Al Bashir Hospital, Amman, Jordan.

**Keywords:** case report, connexin 26 mutation, *GJB2*-related disorders, keratitis-ichthyosis-deafness syndrome, musculoskeletal abnormalities, neurodevelopmental delay, ventriculomegaly in ectodermal dysplasia

## Abstract

**Rationale:**

Keratitis-ichthyosis-deafness (KID) syndrome is a rare ectodermal disorder caused by pathogenic mutations in GJB2 gene, which encodes the gap junction protein connexin 26. While the condition is traditionally defined by a triad of keratitis, ichthyosis, and sensorineural hearing loss, emerging evidence suggests that connexin 26 dysfunction may lead to broader systemic involvement. This case highlights a rare presentation with neurological and musculoskeletal abnormalities.

**Patient concerns:**

A 3-year-old female born at 31 weeks of gestation presented with a history of global developmental delay, recurrent seizures, photophobia, and thick hyperkeratotic skin changes. At birth, she was encased in a collodion membrane and exhibited bilateral eyelid malposition. Her development was marked by delayed milestones, joint stiffness, and poor weight gain.

**Diagnoses:**

Clinical findings included vascularizing keratitis, lamellar ichthyosis, and right-sided sensorineural hearing loss confirmed by auditory brainstem response testing. Brain imaging revealed moderate enlargement of the cerebral ventricles, and skeletal surveys demonstrated developmental dysplasia of the hip and congenital muscular torticollis. A clinical diagnosis of KID syndrome was made based on the constellation of cutaneous, auditory, neurological, and musculoskeletal abnormalities. While genetic testing was unavailable, the phenotype was strongly suggestive of a pathogenic *GJB2* mutation. Although KID syndrome is most commonly caused by autosomal dominant, frequently de novo, mutations-particularly the D50N variant-the apparent autosomal recessive pattern in this pedigree may reflect parental mosaicism, reduced penetrance, or variable expressivity.

**Interventions:**

The patient received coordinated multidisciplinary care. Dermatologic management involved intensive emollient therapy. Ophthalmologic care included lubricants and surgical correction of eyelid malposition. Antiepileptic medication was initiated for seizure control. Physical therapy addressed joint contractures and improved motor function.

**Outcomes:**

Following early intervention, dermatologic symptoms stabilized, seizure activity diminished, and gradual improvements in physical function were observed. However, developmental delay and structural brain abnormalities persisted, requiring long-term follow-up and therapy. Parental compliance multidisciplinary care were essential for optimizing care.

**Lessons:**

This case highlights potential atypical manifestations of KID syndrome, including seizures, ventriculomegaly, torticollis, and hip dysplasia, that may reflect a broader but under-recognized phenotypic range.

## 1. Introduction

Keratitis-ichthyosis-deafness (KID) syndrome is a rare genodermatosis characterized by ectodermal abnormalities affecting the skin, eyes, and inner ear. It is most often associated with missense mutations in the GJB2 gene encoding connexin 26 (Cx26), a gap junction protein critical to intercellular signaling.^[1]^ Pathogenic *GJB2* variants disrupt hemichannel conductance and calcium homeostasis, leading to cellular dysfunction and widespread ectodermal abnormalities. Among these, the D50N mutation is most frequently associated with KID syndrome. Less common variants include G12R (p.Gly12Arg), S17F (p.Ser17Phe), D50Y (p.Asp50Tyr), I30N (p.Ile30Asn), and G11E (p.Gly11Glu), while G45E and A88V mutations have been linked to lethal neonatal presentations.^[1]^

Clinically, KID syndrome is classically defined by a triad of vascularizing keratitis, ichthyosiform dermatosis, and sensorineural hearing loss (SNHL).^[2]^ Ophthalmic manifestations often include progressive photophobia and corneal neovascularization, which may ultimately lead to significant visual impairment.^[1–3]^ Dermatologic features typically present within the first year of life and include thick, hyperkeratotic plaques, palmoplantar keratoderma, ichthyosiform scaling, alopecia, and nail dystrophy.

Although KID syndrome shares phenotypic overlap with other inherited keratinization disorders, it is distinguished by its molecular etiology and systemic features. For instance, autosomal recessive congenital ichthyoses (ARCI) – a heterogeneous group encompassing lamellar ichthyosis (LI), congenital ichthyosiform erythroderma, and harlequin ichthyosis- are characterized by generalized scaling and, often, the presence of a collodion membrane at birth. These conditions typically result from mutations in genes involved in lipid metabolism and epidermal barrier function, such as *TGM1*, *ABCA12*, and *ALOX12B*.^[4]^ While most cases of LI present with a collodion membrane, rare phenotypic variants lacking this hallmark feature have been documented.^[5]^ Importantly, ARCI disorders generally lack both SNHL and keratitis- 2 key features of KID syndrome. This early overlap in presentation underscores the need for genetic testing in neonates with congenital ichthyosis.

Hearing loss affects approximately 1 in 650 newborns and represents the most common congenital sensory deficit.^[6]^ While the majority of cases are non-syndromic, up to 30% occur as part of broader genetic syndromes, including KID. Cx26 is vital for potassium ion recycling within the cochlea, and *GJB2* mutations have been implicated in both syndromic and non-syndromic forms of deafness.^[6]^ Of over 100 identified *GJB2* mutations, only a small subset-including D50N-are associated with syndromic phenotypes marked by hyperkeratosis, hearing loss, and ocular involvement.^[7]^

Emerging evidence suggests that mutations such as D50N may contribute to broader multisystem involvement. Aberrant hemichannel activity in mutant Cx26 proteins has been described as “leaky,” allowing pathological ion flow across the plasma membrane, which can disrupt cellular homeostasis and induce neurotoxicity, apoptosis, and structural brain abnormalities.^[7]^

In this report, we present a case of KID syndrome in a female infant with hallmark dermatologic and auditory features, as well as global developmental delay, seizures, and moderate ventriculomegaly. While isolated ventriculomegaly is not always predictive of neurodevelopmental impairment, its coexistence with seizures and developmental delay raises concern for early central nervous system involvement. This case highlights the expanding phenotypic spectrum of KID syndrome and underscores the importance of early diagnosis, multidisciplinary management, and awareness of potential neurological and musculoskeletal manifestations in affected individuals.

## 2. Case presentation

### 2.1. Perinatal and family history

A 3-year-old female presented to the pediatric outpatient clinic with vomiting, diarrhea, and fever. Her past medical history was significant for a clinical diagnosis of KID syndrome since birth. She is the daughter of a healthy, consanguineous Jordanian couple (first-degree cousins), aged 21 (mother) and 31 (father). Her older sister had similar but milder dermatologic symptoms and died at 2 months of age due to sepsis from acute skin infection. The pregnancy and delivery of the deceased sibling were otherwise uneventful, with a birth weight of 2 kg.

The father reported a family history of dermatologic disorders among second-degree relatives (uncle, grandfather, cousin) with suspected acquired ichthyosis. A family pedigree suggested an autosomal recessive inheritance pattern, supported by the presence of a similarly affected sibling and a history of dermatologic conditions in second-degree relatives (Fig. 3B).

**Figure 1.**
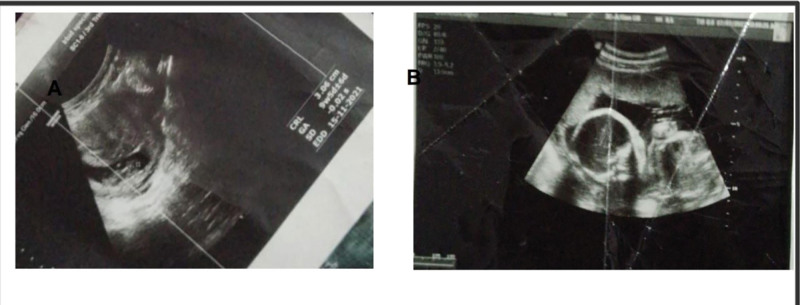
Prenatal ultrasound at 31 wk gestation. (A) Longitudinal fetal lie with unstable positioning. (B) Cross-sectional image showing transverse position. These findings contributed to the decision for cesarean delivery. Preterm birth, along with low birth weight (800 g), increases the risk of neurodevelopmental delay and structural abnormalities, including ventriculomegaly and skeletal anomalies.^[8–10]^

**Figure 2.**
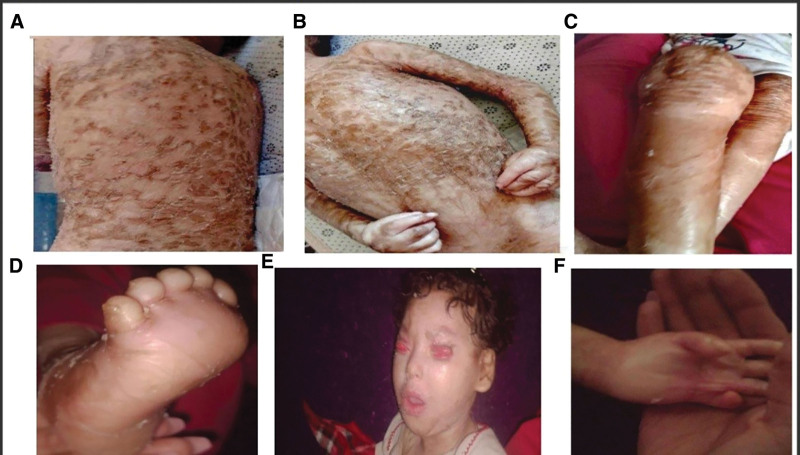
Cutaneous, craniofacial, and musculoskeletal findings in a patient with KID syndrome. (A) Dark brown, thick, plate-like hyperkeratotic scales over the back. (B) Similar scales on anterior chest and upper limbs. (C) Shiny, brown-yellow skin with fissuring at large joints (knees, elbows), indicating impaired mobility. (D) Thin, flaky hyperkeratosis over the dorsum of the foot. (E) Bilateral upper eyelid ectropion, shortened lower eyelids, flattened nasal bridge, and eclabium. (F) Claw hand deformities and pseudocontractures due to skin tightening, limiting joint range of motion.

However, most cases of KID syndrome arise from autosomal dominant, often de novo, mutations in the *GJB2* gene-particularly the D50N variant. Although the pedigree in this case suggests an autosomal recessive pattern, this may instead reflect a dominant *GJB2* mutation with parental mosaicism, reduced penetrance, or variable expressivity. In the absence of molecular testing, we acknowledge that these interpretations remain speculative.^[7]^ Whole exome sequencing was offered to the family; however, they declined due to financial limitations. As such, molecular confirmation of a GJB2 mutation was not possible. Nonetheless, the diagnosis of KID syndrome was based on the classical triad of vascularizing keratitis, ichthyosis (noted after shedding of the collodion membrane), and right-sided sensorineural hearing loss confirmed by auditory brainstem response (ABR) testing. These hallmark features are highly characteristic of KID syndrome and helped guide diagnosis in the absence of genetic testing.

The current patient was delivered via cesarean section at 31 weeks of gestation due to an unstable fetal lie (Fig. 1A and B), premature rupture of membranes, and preterm labor. Her birth weight was 800 grams.

### 2.2. Neonatal course and dermatological evolution

At birth, the neonate was entirely encased in a collodion membrane, including the face and scalp, and exhibited bilateral upper eyelid ectropion. She was admitted to the neonatal intensive care unit for 45 days and placed in a high-humidity incubator to facilitate gradual desquamation. Initial feeding was via orogastric tube, later transitioned to nasogastric tube feeding. At discharge, her weight had increased to 1700 g.

Two weeks post-discharge, the membrane had fully desquamated, revealing thick, dark brown, plate-like hyperkeratotic scales, predominantly involving the back, limbs, and large joints (Figs. 2A–C). Thin, flaky hyperkeratosis was also observed on the face, scalp, and extremities (Fig. 2D), with relative sparing of the face and abdomen. The patient exhibited heat intolerance requiring frequent exfoliation and had rapid nail growth, necessitating regular trimming. The mother also reported photophobia and delayed tooth eruption, with dentition beginning at age 2.

### 2.3. Neurological and developmental assessment

The patient exhibited global developmental delay. Gross motor delays included the inability to crawl, walk, or grasp objects. Fine motor development was absent. Language development was limited to incomprehensible vocalizations, and social interaction was restricted to smiling and crying.

At 4 months of age, she began experiencing generalized tonic-clonic seizures, some of which were febrile in nature. She was admitted to the intensive care unit and received intravenous immunoglobulin. Her immunizations were up to date, except for Bacillus Calmette–Guérin, which was withheld during her NICU stay. She was bottle-fed PediaSure™ and consumed yogurt snacks; emulsified foods were discontinued due to severe constipation.

### 2.4. Physical examination

On examination, the patient exhibited bilateral upper eyelid ectropion, shortened lower eyelids, a flat nasal bridge, eclabium (fish-like mouth), and hypodontia (Fig. 2E). Her skin was parchment-like with a shiny brown hue and prominent fissuring, particularly at joint and flexural areas (Fig. 2C). Claw hand deformities and pseudocontractures were noted, resulting in joint stiffness and limited mobility (Fig. 2F). Deep tendon reflexes were absent. The limbs were normally formed with an appropriate number of digits.

### 2.5. Investigations and diagnosis

**Figure 3.**
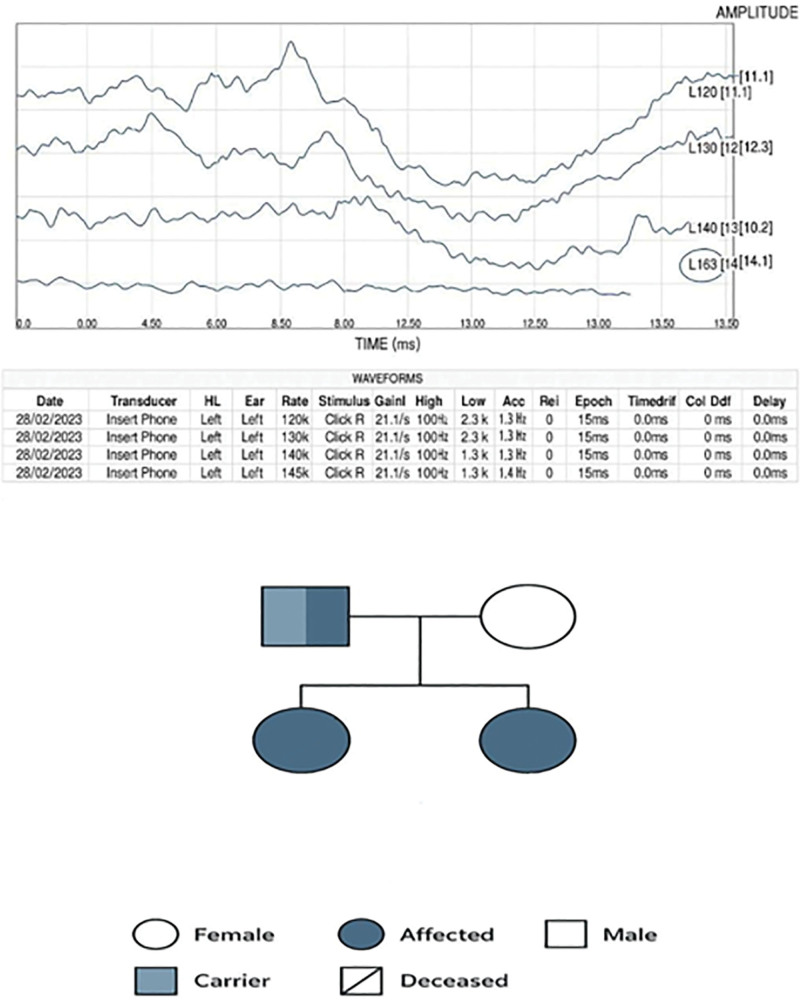
(A) ABR test. Wave I–IV are within normal latency range, but Wave V is delayed, indicating impaired upper brainstem conduction consistent with SNHL. (B) Family pedigree: Shows consanguinity and autosomal recessive inheritance. One affected sibling died from sepsis; additional second-degree relatives had ichthyosis. ABR = auditory brainstem response.

ABR testing confirmed SNHL (Fig. 3A). An electroencephalogram (EEG) performed at 4 months of age showed no epileptiform activity; however, due to ongoing seizure-like episodes reported by both family members and healthcare providers, antiepileptic therapy with Levetiracetam was initiated.

**Figure 4.**
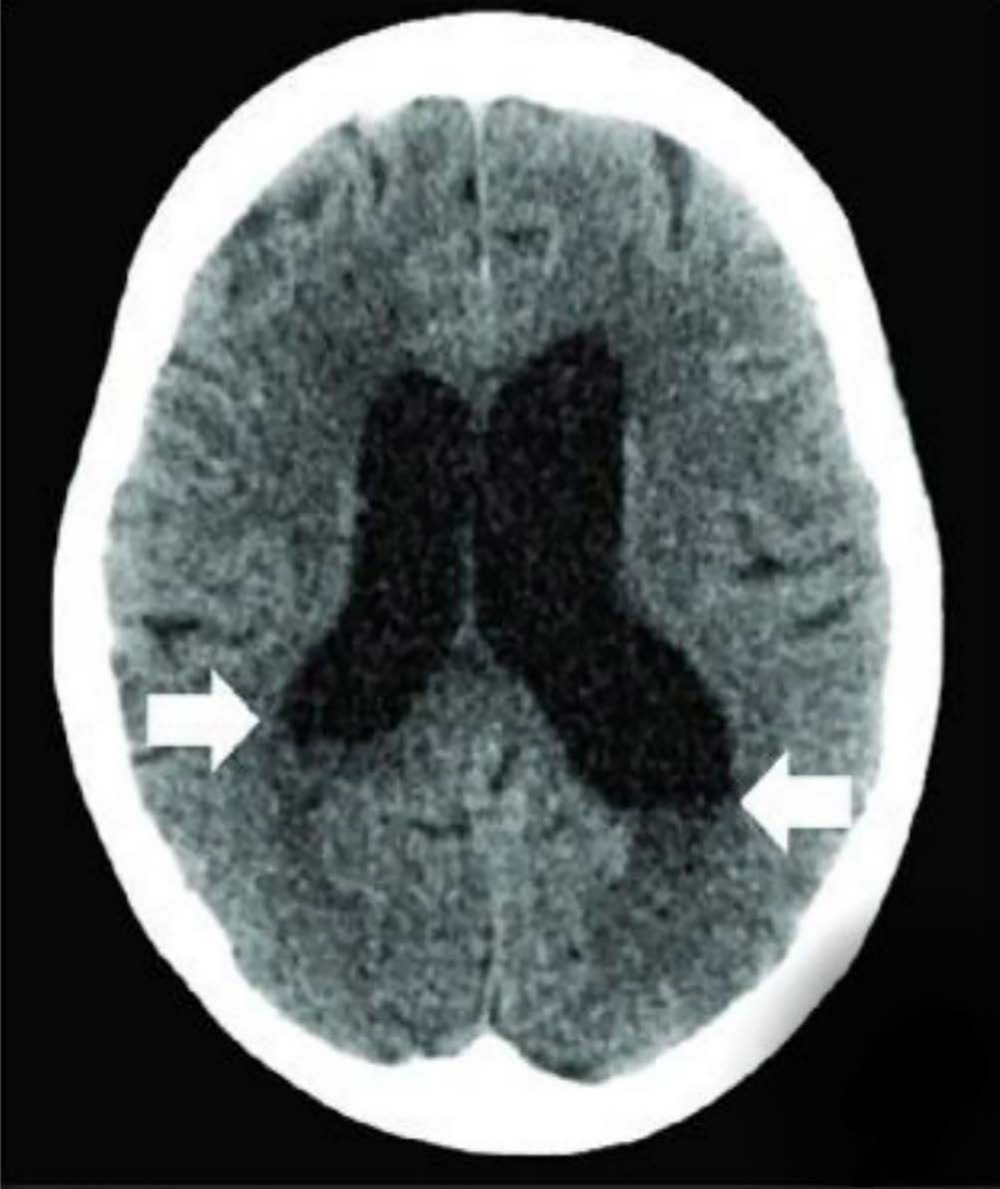
Axial CT image demonstrating ventricular dilation. White arrows indicate frontal horn width. The FOHR, calculated from this image, was estimated at approximately 0.5, consistent with moderate ventriculomegaly. CT = computerized tomography, FOHR = fronto-occipital horn ratio.

At 2 years and 4 months of age, a non-contrast brain computed tomography (CT) scan revealed ventricular dilation without evidence of hemorrhage, midline shift, calcifications, or structural malformations (Fig. 4). In this case, the degree of ventricular dilation was assessed using the axial CT image. To quantify this finding, we applied the fronto-occipital horn ratio (FOHR), a validated and widely used linear metric for evaluating ventriculomegaly in pediatric neuroimaging. Based on approximate visual estimation from the submitted scan (Fig. 4), the FOHR was calculated at ~0.5, corresponding to moderate ventriculomegaly per established thresholds (FOHR ≥ 0.5). This method was selected due to its simplicity and reproducibility on routine axial CT, without requiring volumetric MRI or post-processing software.

During this visit, it was also noted that the patient’s mother had discontinued antiepileptic medication on multiple occasions due to the absence of seizures. Upon resuming treatment, liver function tests revealed a mild elevation in aspartate transaminase (AST) levels (43 U/L; normal ≤ 37 U/L).

**Figure 5.**
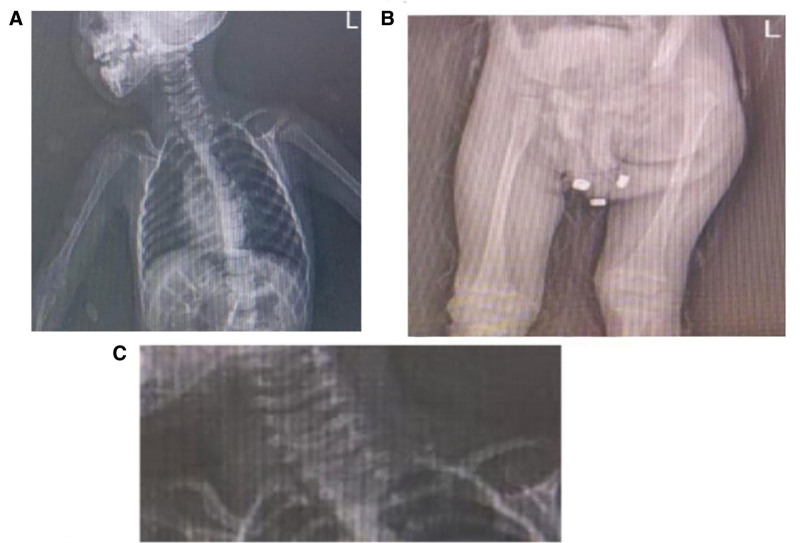
(A) Upper torso and shoulder X-ray showing malformed clavicle, elevated right scapula, and delayed ossification of ribs and vertebrae. (B) Pelvic imaging demonstrates DDH, with misalignment of the femoral head and acetabulum. (C) Lateral cervical spine X-ray reveals congenital muscular torticollis with misalignment of C1 and C2 vertebrae, suggestive of neuromuscular imbalance. DDH = developmental dysplasia of the hip.

Skeletal surveys demonstrated findings consistent with torticollis and delayed bone development, particularly involving the clavicles, hips, and cervical spine (Fig. 5A–C).

### 2.6. Management and follow-up

The patient received multidisciplinary care from dermatology, ophthalmology, audiology, neurology, and pediatrics. Dermatologic management included topical emollients (glycerin, petrolatum, liquid paraffin). Ophthalmic care involved lubricating eye drops (hydroxypropyl methylcellulose and dextr 70) and surgical correction of eyelid malposition at 6 months via skin grafting from the upper arm. Due to inadequate primary closure, the donor site was allowed to heal by secondary intention.

Antiepileptic therapy with Levetiracetam was continued with liver function monitoring. Neurological follow-up was maintained for seizure control and developmental support. Moreover, the patient began physical therapy targeting joint contractures and reduced flexibility due to skin tightening. The program included stretching, massage, and strengthening exercises, which improved her mobility and comfort.

A chronological summary of the patient’s clinical milestones, interventions, and associated rationale is provided in Table 1. This timeline highlights the evolution of her condition and the multidisciplinary approach to her management.

**Table 1:** Timeline of clinical events with associated rationale.

Age	Event	Purpose/rationale
Birth (31 wk)	Preterm delivery due to PROM; NICU stay; 45 d in humidified incubator; collodion membrane noted; birth weight 800 g	Manage complications of prematurity and ectodermal dysplasia; monitor growth, hydration, and skin health
2 wk	Collodion membrane shed; dark-brown hyperkeratosis noted on trunk and limbs	Establish dermatological diagnosis; initiate emollient-based skin care
4 mo	2–3 seizures/mo (febrile and afebrile); EEG normal; Levetiracetam started at 10 mg/kg/d, titrated to 20 mg/kg/d	Evaluate neurological involvement; initiate seizure control with AED monotherapy
6 mo	Surgical correction of bilateral ectropion with skin graft from upper arm	Prevent corneal exposure; preserve visual function
1–2 yr	Seizure frequency decreased to 1–2/mo; Levetiracetam maintained; patient seizure-free for ~5–6 mo before medication nonreported	Monitor treatment response; support caregiver adherence; adjust dose with weight gain
2 yr 4 mo	Medication intermittently stopped by caregiver; abnormal febrile movements reported; CT showed moderate ventriculomegaly (FOHR ≈ 0.5); AST = 43 U/L	Assess global developmental delay; monitor for AED side effects; resume Levetiracetam with LFT monitoring
3 yr	No tonic-clonic seizures in 11 mo; Levetiracetam 20 mg/kg/d (~210 mg/d); developmental delay persists; PT initiated	Maintain seizure control; enhance mobility and quality of life through rehab and multidisciplinary care

A chronological overview of the patient’s key clinical milestones, diagnostic investigations, therapeutic interventions, and follow-up outcomes. The table summarizes the purpose of each step in management and highlights the multidisciplinary approach used throughout the patient’s care.

CT = computerized tomography, EEG = electroencephalogram, FOHR = fronto-occipital horn ratio

## 3. Discussions

### 3.1. Clinical presentation and diagnostic overview

#### 3.1.1. Initial presentation

This case report presents a rare and severe phenotype of KID syndrome in a 3-year-old female born prematurely at 31 weeks gestation. At birth, she exhibited a parchment-like collodion membrane, a hallmark of ARCI, particularly LI. Additional complications such as hyperthermia, dehydration, and constriction bands were present, indicating a disrupted skin barrier and systemic involvement from early postnatal life.^[8,9]^ These features point to extensive dysregulation in ectodermal development, necessitating a high index of suspicion for syndromic forms.

In addition to the classical cutaneous findings, our patient exhibited multiple atypical features, including ventriculomegaly, recurrent seizures, developmental delays, congenital torticollis, and DDH. These findings, not commonly emphasized in standard KID syndrome presentations, suggested a broader multisystem involvement and prompted further multidisciplinary evaluation.

#### 3.1.2. Diagnostic workup

A multidisciplinary evaluation supported the diagnosis based on key clinical and radiographic findings. ABR confirmed right-sided SNHL. Ocular examination revealed signs of keratitis, and brain magnetic resonance imaging (MRI), conducted in response to developmental delays and recurrent seizures, showed moderate ventriculomegaly. The differential diagnosis initially included other forms of ARCI, notably congenital ichthyosiform erythroderma and harlequin ichthyosis; however, the absence of erythroderma or plate-like scales argued against those phenotypes. The combination of ichthyosis following collodion membrane desquamation, vascularizing keratitis, and unilateral sensorineural hearing loss represents the hallmark triad of KID syndrome and guided the clinical diagnosis in this case. Although molecular confirmation via GJB2 sequencing was not pursued due to financial limitations, the phenotype closely aligns with syndromic presentations associated with pathogenic connexin 26 variants, particularly the D50N mutation. In light of these findings and emerging evidence linking GJB2 mutations to neurological and skeletal anomalies, this report aims to contribute a hypothesis-generating perspective on the multisystemic manifestations of KID syndrome. Although the pedigree appeared to suggest an autosomal recessive inheritance pattern- due to parental consanguinity and an affected sibling- most reported cases of KID syndrome are caused by autosomal dominant, often de novo, mutations in the *GJB2* gene, particularly the D50N variant.^[10,11]^ This discrepancy underscores the importance of molecular confirmation in syndromic ichthyoses and highlights how consanguinity can mask dominant inheritance by mimicking a recessive patter n.

Given the constellation of systemic features, attention turned to potential molecular underpinnings, particularly those involving connexin proteins.

#### 3.1.3. Molecular insights into Cx26 (GJB2) mutations

The *GJB2* gene encodes connexin 26 (Cx26), a transmembrane protein that forms gap junctions to facilitate direct intercellular communication. The D50N missense mutation is the most frequently associated pathogenic variant in KID syndrome and has been shown in experimental studies to result in constitutively open hemichannels, leading to increased membrane permeability, oxidative stress, and eventual cell death.^[10–13]^ Only a small subset of *GJB2* mutations-including D50N-are associated with syndromic phenotypes characterized by concurrent dermatologic, auditory, and ocular involvement. A comprehensive review by Xiang et al further supports this by mapping the phenotypic spectrum of single-nucleotide *GJB2* substitutions and differentiating syndromic from non-syndromic outcomes.^[14]^ Additional mutations- such as G12R, A88V, and G45E-also contribute to aberrant hemichannel behavior and impair proper gap junction plaque formation. These mutations often impact the cytoplasmic loop and C-terminal domains, which are essential for hemichannel gating and trafficking. Recent studies have demonstrated that pathogenic variants of *GJB2*, localized to specific Cx26 domains, exert distinct functional consequences- reinforcing the importance of domain-specific analysis in clinical interpretation.^[15]^

To further highlight the phenotypic breadth observed in our patient, we reflect on classical features of KID syndrome in contrast to this case and atypical findings reported in the literature. While the triad of ichthyosis, keratitis, and SNHL was consistent, our patient’s unilateral hearing loss, ventriculomegaly, seizures, torticollis, and DDH extend the clinical landscape. These findings align with emerging reports that suggest Cx26 dysfunction may impact neural and mesenchymal tissues, necessitating broader diagnostic consideration.

### 3.2. Pathophysiology and multisystem involvement

While prematurity and consanguinity may contribute to neurological and musculoskeletal findings such as ventriculomegaly, developmental delay, and torticollis, their presence in conjunction with the classical triad of KID syndrome warrants further consideration. Recent literature has associated GJB2 mutations, particularly D50N, with aberrant neurodevelopment and structural brain anomalies. In this context, the patient’s broader phenotype may reflect a syndromic extension of connexin 26 dysfunction. Although causality cannot be definitively established, these findings underscore the need for further research into the multisystem manifestations of KID syndrome.

#### 3.2.1. Cx26 and neurological dysfunction

Cx26 mutations such as D50N have profound effects on neuronal physiology. Disrupted calcium homeostasis, aberrant ATP signaling, and elevated hemichannel activity contribute to increased neuronal excitability and vulnerability to seizures.^[16–22]^ Studies by Richard et al and Kelsell et al support the role of D50N in disrupting neuroepithelial integrity and signal propagation.^[10,23]^ Cx26 expression in both neurons and glial cells implicates it in diverse neurological functions, including synaptic plasticity and network synchronization.

#### 3.2.2. Brain anomalies in KID syndrome

There is increasing recognition of structural CNS anomalies associated with KID syndrome. Cerebellar hypoplasia, reported in other patients with Cx26 mutations, highlights the gene’s potential role in neurodevelopment.^[24]^ Furthermore, diffusion tensor imaging and volumetric MRI in children with GJB2-related deafness have demonstrated altered white matter microstructure and cortical volume changes, suggesting Cx26 disruption may influence neuroanatomical maturation even in the absence of syndromic features.^[25]^ Our patient’s moderate ventriculomegaly may represent an underreported phenotype within this continuum. Previous studies have shown that isolated ventriculomegaly in very low birth weight and very low gestational age infants does not necessarily predict significant neurodevelopmental impairments.^[26]^ However, research- including a 2023 meta-analysis- has demonstrated that preterm infants who experience intraventricular hemorrhage or white matter injury are at increased risk of long-term cognitive and motor deficits.^[27,28]^

#### 3.2.3. Cx26 in glial and synaptic function

Beyond neurons, Cx26 is expressed in astrocytes and oligodendrocytes, where it contributes to extracellular ion regulation and metabolic support.^[29–34]^ Impaired potassium buffering due to dysfunctional astrocytic gap junctions may underlie the patient’s seizure susceptibility and developmental delays. Additionally, Cx26 influences the process of myelination and oligodendrocyte maturation- dysfunction in this area could explain structural brain abnormalities and motor delays. Notably, recent models have showGn that glial-specific deletion of Cx26 leads to behavioral and morphological phenotypes resembling leukodystrophies.^[35]^

#### 3.2.4. Musculoskeletal manifestations and cytoskeletal disruption

Emerging evidence suggests that Cx26 is critical not only for epithelial but also mesenchymal tissue integrity. Our patient’s torticollis, joint stiffness, and DDH may result from altered cytoskeletal dynamics and impaired Cx26 trafficking. Although a direct link between Cx26 and hip joint development remains incompletely defined, gap junction proteins- including Cx26-are implicated in mechanotransduction and cellular differentiation in cartilage and bone. Disrupted intercellular signaling during embryogenesis could impair acetabular formation or joint stability.^[36]^

While some reports suggest Cx26 trafficking is actin-independent,^[37]^ others demonstrate reduced F-actin levels and defective dye coupling with Cx26 knockdown.^[38,39]^ These findings indicate that impaired actin-Cx26 interactions may disrupt cell adhesion, mechanotransduction, and osteoblast/myocyte function.^[40–43]^ Collectively, these mechanisms support a role for Cx26 dysfunction in both neurological and structural musculoskeletal abnormalities.

#### 3.2.5. Integrative pathophysiological model

The mechanisms discussed in this section are derived from in vitro and animal studies of GJB2 mutations and are not directly demonstrated in this case. They are included to provide biological plausibility and to support a hypothesis-generating framework.

The D50N mutation exemplifies a pathogenic cascade initiated by constitutively open hemichannels, resulting in aberrant calcium influx, oxidative stress, and apoptosis.^[13]^ Disruptions in glial signaling, cytoskeletal organization, and gap junction communication converge to produce the multisystemic features observed in KID syndrome. Interactions between Cx26 and actin-binding proteins such as ZO-1 and drebrin regulate tissue cohesion and signaling at the cellular junction level, implicating cytoskeletal disarray in disease pathology.^[44]^

This constellation of dysfunctions, spanning neuronal, glial, epithelial, and mesenchymal tissues, highlights the systemic nature of Cx26-related disease. Foundational and recent studies have mapped the physiological roles of connexins-including Cx26-across neural, epithelial, and cardiovascular systems, reinforcing the view of KID syndrome as a pan-ectodermal and systemic disorder.^[18,45,46]^

Recent imaging and clinical studies have also noted structural abnormalities in GJB2-related conditions. For example, Kenna et al (2011) described inner-ear malformations, including semicircular canal hypoplasia and vestibular aqueduct enlargement, in a subset of children with biallelic GJB2 mutations.^[47]^ Meanwhile, aberrant connexin 26 hemichannels have been mechanistically implicated in neurotoxicity and structural brain dysfunction in animal models of KID syndrome. These emerging insights lend additional plausibility to a broader spectrum of connexin-related tissue involvement and support the consideration of neurological and skeletal features within the extended phenotypic range of KID syndrome.^[22]^

### 3.3. Management and intervention

Early, proactive management was crucial in this case. In the neonatal period, the patient was placed in a humidified incubator to reduce transepidermal water loss (TEWL) and received orogastric, then nasogastric, feeding for nutritional support. Barrier repair therapy with emollients containing glycerin, petrolatum, and paraffin was initiated.^[9,48]^ Ophthalmologic interventions included frequent lubricants and a full-thickness skin graft at 6 months to correct lower eyelid ectropion. Physical therapy focused on neurodevelopmental therapy, range-of-motion exercises, and targeted strength training. These interventions yielded significant improvements in tone and functional mobility.

### 3.4. Multidisiplinary follow-up strategy

- **Dermatologic Care:** Lifelong use of emollients to minimize TEWL and reduce infection risk.
- **Ophthalmologic Care:** Long-term ocular lubrication and surgical correction for eyelid malformations.
- **Audiologic Support:** Unilateral SNHL confirmed by ABR; evaluation for assistive hearing devices.
- **Neurological Management:** Levetiracetam for seizure control, periodic EEG and neuroimaging follow-up.
- **Musculoskeletal Care:** Continued physiotherapy and monitoring for evolving skeletal or motor anomalies.

### 3.5. Prognosis and outcomes

The patient’s prognosis hinges on sustained multidisciplinary involvement. Although her seizures are currently controlled, discontinuation of antiepileptics poses a risk of recurrence. Ventriculomegaly warrants serial monitoring given its potential impact on cognitive outcomes. Physical and developmental progress are promising but depend on ongoing therapy. Dermatologic vigilance is essential for infection prevention and quality of life maintenance. Audiologic and ophthalmologic follow-up are key for sensory development.

### 3.6. Implications and future directions

This case broadens the phenotypic spectrum of KID syndrome, highlighting its characterization as a multisystem connexinopathy. In addition to the classical triad, our patient presented with seizures, ventriculomegaly, torticollis, and DDH-findings that implicate Cx26 in widespread tissue dysfunction, beyond ectodermal derivatives.

Future directions include:

- Expanded neuroimaging and longitudinal developmental assessments in Cx26 mutation carriers.
- In vitro and in vivo studies evaluating hemichannel blockers as therapeutic adjuncts.^[49]^
- Investigation of gene editing tools (e.g., CRISPR-Cas9) to correct D50N and related mutations at the source.
- Systems biology approaches to map the Cx26 interactome and uncover modifiers of disease severity.
- Further exploration of cytoskeletal and glial pathways to identify molecular targets for musculoskeletal and neuroprotective therapies.^[50]^

Altogether, this case underscores the need for a holistic diagnostic lens and personalized care model in patients with suspected or confirmed GJB2 mutations. As outlined in recent consensus literature, GJB2 mutations- particularly those affecting the D50 residue- have been associated with structural protein dysfunction, aberrant hemichannel activity, and diverse phenotypic outcomes depending on tissue-specific expression.^[18,46]^ Incorporating such mechanistic understanding may enhance diagnostic precision and promote the development of mutation-specific therapeutic strategies in the future.

## 4. Conclusion

This case report offers a comprehensive analysis of a rare and severe presentation of KID syndrome, illustrating its potential to affect multiple organ systems beyond the classic triad of keratitis, ichthyosis, and deafness. Our patient exhibited not only dermatological and auditory symptoms but also significant neurological and musculoskeletal involvement, including seizures, global developmental delay, torticollis, and hip dysplasia. These findings add to the growing recognition of phenotypic variability associated with Cx26 mutations and may contribute to a more nuanced understanding of syndromic presentations, particularly in cases where classical features coexist with atypical neurological and skeletal findings.

The observation of moderate ventriculomegaly adds to the underexplored neurodevelopmental spectrum of KID syndrome. This anomaly, in conjunction with early-onset seizures and developmental delay, emphasizes the need for ongoing neurological evaluation in affected patients. Coupled with the suspected role of Cx26 in neuronal signaling and cytoskeletal organization, our findings offer new insights into the broader pathophysiology of the disorder.

Importantly, the patient’s improvement with timely multidisciplinary interventions- including emollient therapy, surgical correction, physiotherapy, and seizure management- demonstrates the effectiveness of early, integrative care. This reinforces the importance of prompt diagnosis, close surveillance, and personalized treatment strategies.

By documenting a complex and atypical presentation, this report contributes to the limited literature on KID syndrome and supports an emerging consensus that Cx26 mutations may underlie a broader, multisystemic disorder. This case illustrates the importance of investigating the neurological and musculoskeletal implications of Cx26 mutations and calls for further studies to explore molecular mechanisms linking connexin dysfunction to CNS and skeletal development. Although genetic testing was not pursued due to financial limitations, the patient’s phenotype, including the full triad of keratitis, ichthyosis, and SNHL, was strongly suggestive of KID syndrome. We present this case to highlight the potential for syndromic diagnosis based on phenotype and to encourage further research on the neurological spectrum of GJB2 mutations. The findings may ultimately guide more holistic diagnostic frameworks and targeted therapeutic innovations across the spectrum of connexinopathies.

To further contextualize the observed phenotype, we present Table 2, which compares classical features of KID syndrome with those observed in our patient and with atypical findings reported in the literature. This visual summary underscores the potential for broader, under-recognized manifestations of GJB2 mutations and supports the need for a more expansive diagnostic framework when evaluating patients with suspected syndromic ichthyosis.

**Table 2:** Comparison of classical and atypical KID syndrome features in the present case 638 and published literature.

Features	Classical KID syndrome	This case	Previous publications	References
Ichthyosis	Present	Present (collodion membrane)	Common in all variants	[13,14]
Keratitis	Present	Present	Consistent	[13,14]
Sensorineural hearing loss	Present	Unilateral	Usually bilateral	[6,13,14]
Seizures	Rarely reported	Present	Emerging evidence	[13,16,20–26]
Ventriculomegaly*	Not typically reported	Present	Occasion (e.g. cerebral hypoplasia)	[27,28,43]
Developmental delay	Occasional	Present	Associated with severe cases	[16,28,43]
Torticollis*	Not reported	Present	Very limited data	[13]
DDH*	Not reported	Present	Rarely described	[13,15]
Genetic confirmation	Typically confirmed (GJB2 mutation)	Not performed	Often confirmed	[13,18]
Histopathology	Variable	Not performed	Rare but useful	[44]

This table outlines key clinical features typically reported in classical 639 KID syndrome, compared to the manifestations observed in our patient and those described in 25 640 atypical presentations. The inclusion of neurological and musculoskeletal findings in our case 641 contributes to the growing recognition of KID syndrome as a multisystem connexinopathy.

Features marked with an asterisk (

* ) are not commonly associated with classical KID syndrome and may be incidental or underreported. They are presented here for hypothesis-generating comparison in light of evolving phenotypic descriptions in GJB2-related disorders.

DDH = developmental dysplasia of the hip, GJB2 = gap junction beta-2, KID = keratitis-ichthyosis-deafness

## Author contributions

**Conceptualization:** Bayan K. AlRababah, Yara Abukhaled, Gharam Ghalyon, Anas Satari.

**Data curation:** Bayan K. AlRababah, Miral S. Abu Rumman, Hamzeh K. Bany Younis, Muhanad Maaita, Anas Satari.

**Investigation:** Bayan K. AlRababah, Yara Abukhaled, Safaa Al-Tawalbeh, Aya Khaled D. Salah.

**Methodology:** Rama Al-Bustanji.

**Project administration:** Rama Al-Bustanji, Miral S. Abu Rumman.

**Resources:** Bayan K. AlRababah, Yara Abukhaled, Nizar Al-Rabadi, Hamzeh K. Bany Younis, Gharam Ghalyon, Mu‘nis Muneeb Mohammad Alrashdan, Mohannad Thafer Yamin, Muhanad Maaita, Aya Khaled D. Salah.

**Supervision:** Rama Al-Bustanji, Safaa Al-Tawalbeh, Anas Satari.

**Validation:** Yara Abukhaled, Safaa Al-Tawalbeh, Osama Aloudat, Mohannad Thafer Yamin.

**Visualization:** Hamzeh K. Bany Younis.

**Writing – review & editing:** Rama Al-Bustanji, Miral S. Abu Rumman, Yara Abukhaled, Nizar Al-Rabadi, Hamzeh K. Bany Younis, Gharam Ghalyon, Safaa Al-Tawalbeh, Osama Aloudat, Mu‘nis Muneeb Mohammad Alrashdan, Mohannad Thafer Yamin, Muhanad Maaita, Aya Khaled D. Salah, Anas Satari.

**Writing – original draft:** Rama Al-Bustanji, Bayan K. AlRababah, Miral S. Abu Rumman, Nizar Al-Rabadi.
